# Formulation and Evaluation of Different Nanogels of Tapinarof for Treatment of Psoriasis

**DOI:** 10.3390/gels10110675

**Published:** 2024-10-22

**Authors:** Barbara Balogh, Ágota Pető, Ádám Haimhoffer, Dávid Sinka, Dóra Kósa, Pálma Fehér, Zoltán Ujhelyi, Monica Argenziano, Roberta Cavalli, Ildikó Bácskay

**Affiliations:** 1 Department of Pharmaceutical Technology, Faculty of Pharmacy, University of Debrecen, Nagyerdei Körút 98, H-4032 Debrecen, Hungary; balogh.barbara@pharm.unideb.hu (B.B.); peto.agota@pharm.unideb.hu (Á.P.); haimhoffer.adam@pharm.unideb.hu (Á.H.); sinka.david@pharm.unideb.hu (D.S.); kosa.dora@pharm.unideb.hu (D.K.); feher.palma@pharm.unideb.hu (P.F.); ujhelyi.zoltan@pharm.unideb.hu (Z.U.); 2 Doctoral School of Pharmaceutical Sciences, University of Debrecen, Nagyerdei Körút 98, H-4032 Debrecen, Hungary; 3 Institute of Healthcare Industry, University of Debrecen, Nagyerdei Körút 98, H-4032 Debrecen, Hungary; 4 Department of Drug Science and Technology, University of Turin, Via P. Giuria 9, 10125 Turin, Italy; monica.argenziano@unito.it (M.A.); roberta.cavalli@unito.it (R.C.)

**Keywords:** tapinarof, nanogels, psoriasis, SNEDDS, wound healing

## Abstract

Psoriasis is an immune-mediated skin condition. There are many therapeutic options for its treatment; however, none of them is completely effective. Tapinarof is a promising new active substance; it was recently approved by the FDA for the treatment of psoriasis. The aim of our study was to prepare topical nanoformulations of the active substance to improve its bioavailability and therapeutic effect. The biocompatibility investigation of the formulations was carried out by MTT assay, and the size distribution of the preparations was investigated by DLS. In parallel, the rheological properties and the texture were examined, and the in vitro release of tapinarof was assessed by the Franz diffusion method. An in vitro wound healing assay was also carried out to study the drug’s properties. Our results show that the preparations were safe on HaCaT cells. The particle size of the formulations was proven to be in the nanoscale range. In the in vitro release studies, nanogel II. showed greater release of the active substance. According to the wound healing assay, incorporation of the drug into nanoformulations increases the antiproliferative and antimigration activity of the drug. Overall, nanoformulations of tapinarof were successfully prepared, and our results suggest that they can be a useful addition to the current therapeutic practice of psoriasis.

## 1. Introduction

Psoriasis is a chronic, immune-mediated skin disorder associated with inflammation in the body due to immune dysfunction that affects 1–3% of the world’s population [1,2,3]. This condition is associated with persistent inflammation and excessive growth of skin cells [2]. The disease is characterized by alternate periods of remission and exacerbation [1,4]. The most common type of psoriasis is plaque psoriasis (psoriasis vulgaris). Visible signs on the skin can appear in the form of pruritic, well-defined lesions, plaques, and scales; that is where the name originates from [1,5]. In addition to skin symptoms, several significant comorbidities are related to psoriasis, including cardiovascular disease, psoriatic arthritis, metabolic syndrome, obesity, inflammatory bowel disease, and major depression [5,6]. Most patients have mild, localized lesions and do not require systemic treatment. Local therapy is crucial in the treatment of these patients. Unfortunately, there is no current cure; however, by reducing the symptoms, we can improve the quality of life of the patients. Currently, available topical therapies include vitamin D analogs (calcipotriene or calcitriol), vitamin A analogs (tazarotene), corticosteroids, and coal tar. However, these have a number of side effects that limit their use in the short- and long-term, meaning that the appropriate topical treatment of psoriasis remains an important unresolved issue [1,7,8,9,10].

Tapinarof is a new topical therapeutic agent approved in China in 2019 and in the United States in 2022 for the topical treatment of psoriasis in adults [4,7,11,12]. It is the first and only licensed non-steroidal topical medicine for this condition [2,3,7,8,10,13]. Susan H. Smith et al. proved that a one-of-a-kind small molecule was shown to have anti-inflammatory characteristics [14].

The effect of tapinarof in psoriasis is related to the activation of the aryl hydrocarbon receptor (AhR), a ligand-dependent transcription factor that regulates gene expression in a variety of cells, including immune and epithelial cells. This leads to decreased skin inflammation by downregulation of the psoriasis-associated cytokines IL-17A, IL-17F, and IL-22. It also normalizes the skin barrier through increased expression of skin barrier proteins associated with keratinocyte differentiation, including filaggrin, loricrin, and involucrin. The molecule is able to reduce oxidative stress via increased antioxidant response through the Nrf2 pathway [2,3,5,7,8,11,12,13,15].

The main problem with transdermal drug delivery is the barrier properties of the stratum corneum, which is considered one of the most impermeable epithelial layers of the human body to exogenous substances. In the case of psoriasis, the skin barrier is damaged, and therefore the penetration of active substances is reduced. These permeation problems can be minimized by using chemical permeation enhancers. However, their use can be harmful, especially in chronic conditions, as many of them tend to be irritating [16,17]. Moreover, about 40% of new chemical agents are hydrophobic in nature, such as tapinarof. The delivery of these poorly water-soluble agents also challenges drug delivery [16,18,19]. Therefore, it is desirable to develop a new transdermal delivery system that does not require chemical enhancers to promote the permeation of drugs through the skin. [16,17]

In the last decade, with the development of nanotechnology, nanocarriers have emerged and gained popularity in biomedicine. A number of studies have investigated the potential of nanoemulsions for topical drug delivery, with the recognition that they behave differently when interacting with biological barriers [18,19]. Emulsions and nanoemulsions differ mainly in the size and shape of the particles dispersed in the continuous phase. The particle size of nanoemulsions is 10–200 nm, compared to conventional emulsions, which have a particle size of 1–20 μm. Nanoemulsions offer a wide range of possibilities for topical drug delivery, but their low viscosity limits their use in transdermal applications due to their cumbersome application. A variety of gelling agents, such as carbomer 940 and carbomer 934 have been used to increase their viscosity. Consequently, the incorporation of nanoemulsions into a gel matrix results in nanogels, which are more relevant for topical application [16]. They are characterized by three-dimensional hydrophilic polymeric network structures of nanoscale sizes with high stability and spreadability [17,19].

Tapinarof is currently available in the form of cream on the market [11,12,20]. However, the transdermal permeation and the bioavailability of the active substances can be further enhanced with nanogel formulations, which have numerous advantages over traditional ointments and creams. Their unique physicochemical properties enable improved drug solubility and transdermal enhancement. Furthermore, the gel as a topical formulation hydrates the skin surface and supports increased skin permeability of the drugs, which increases patients’ compliance [19,21,22].

Since psoriasis is a challenging disease with symptoms (itching, redness, and peeling) that have a significant influence on the quality of life of patients. The aim of our study was to formulate innovative nanogel formulations containing tapinarof to keep the patient in recovery or remission for longer, potentially reducing the need for biological therapies. Our motivation was based on numerous examples in the literature where the skin permeability and bioavailability of several active ingredients, such as ibuprofen, ketoprofen, and curcumin were successfully enhanced using nanocarrier-based systems [23,24,25]. Tapinarof is a revolutionary drug that could open up new perspectives in the treatment of psoriasis. Research on tapinarof-containing nanogels is particularly promising, as the use of nanotechnology allows the active ingredients to target directly and effectively the problem areas in the skin. This approach can not only increase the effectiveness of the treatment but also reduce side effects (headache, skin rash, irritation) [17,20]. In the formulation process, different nanogel formulations were prepared and tested from different aspects such as size analysis, biocompatibility, texture, and rheology, as well as wound healing capacity [16].

## 2. Results and Discussion

### 2.1. Droplet Size Distribution

**Figure 1:**
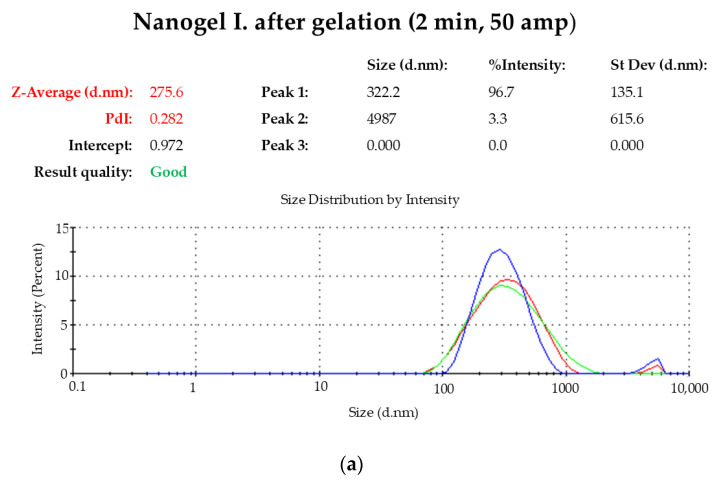

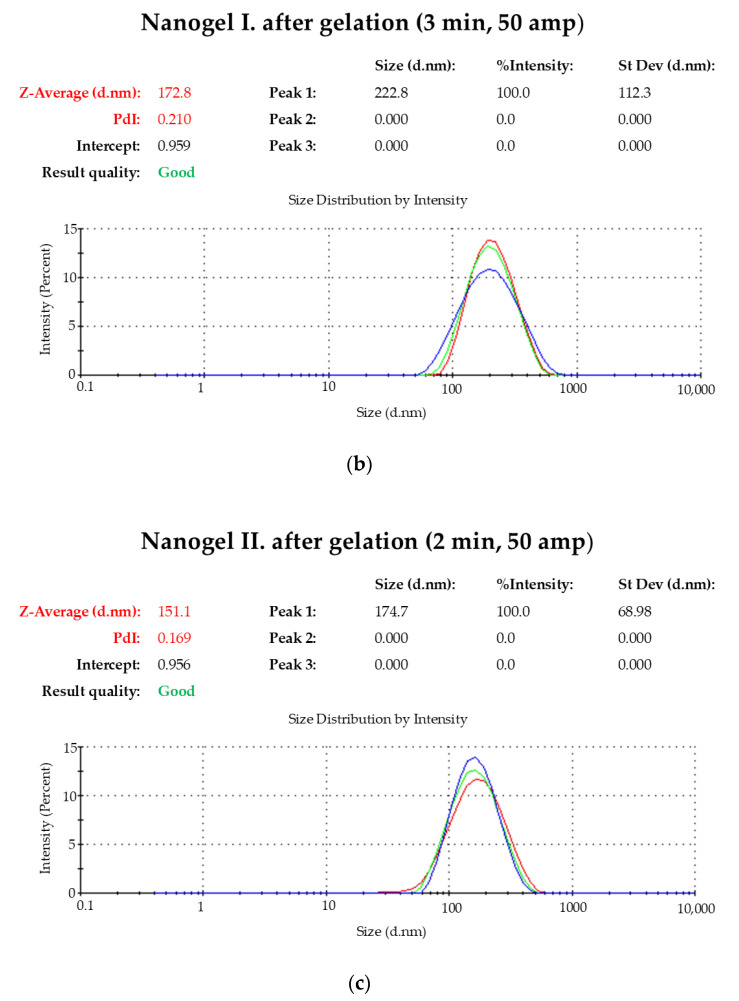
(**a**–**c**) Droplet size distribution of the preparations.

The distribution of droplet size and polydispersity index of both compositions were determined and presented in Figure 1. According to the DLS measurements, both compositions are in the nano-size range. To achieve this range, the gels were subjected to manual ultrasonic treatment before gelation. In the case of gel II., the application of 50 amplitudes for 2 min was sufficient to achieve the required nano-size, which was maintained after gelation. This time was not enough for gel I. as it is illustrated in Figure 1. For this reason, the time was increased to 3 min, resulting in nano-sized gels. In addition, all polydispersity indexes of the formulations were found in the low values. Summary of data are summarized in Table 1 and Table 2.

### 2.2. Cell Viability Study

**Figure 2:**
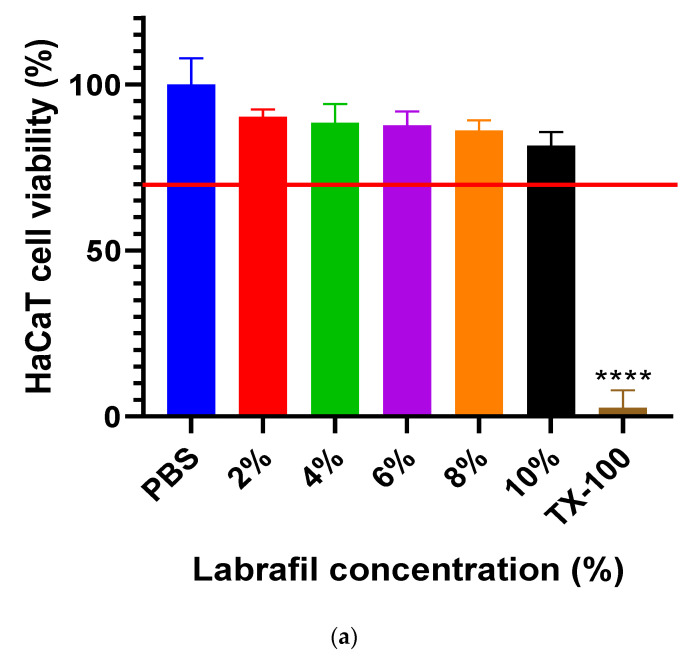

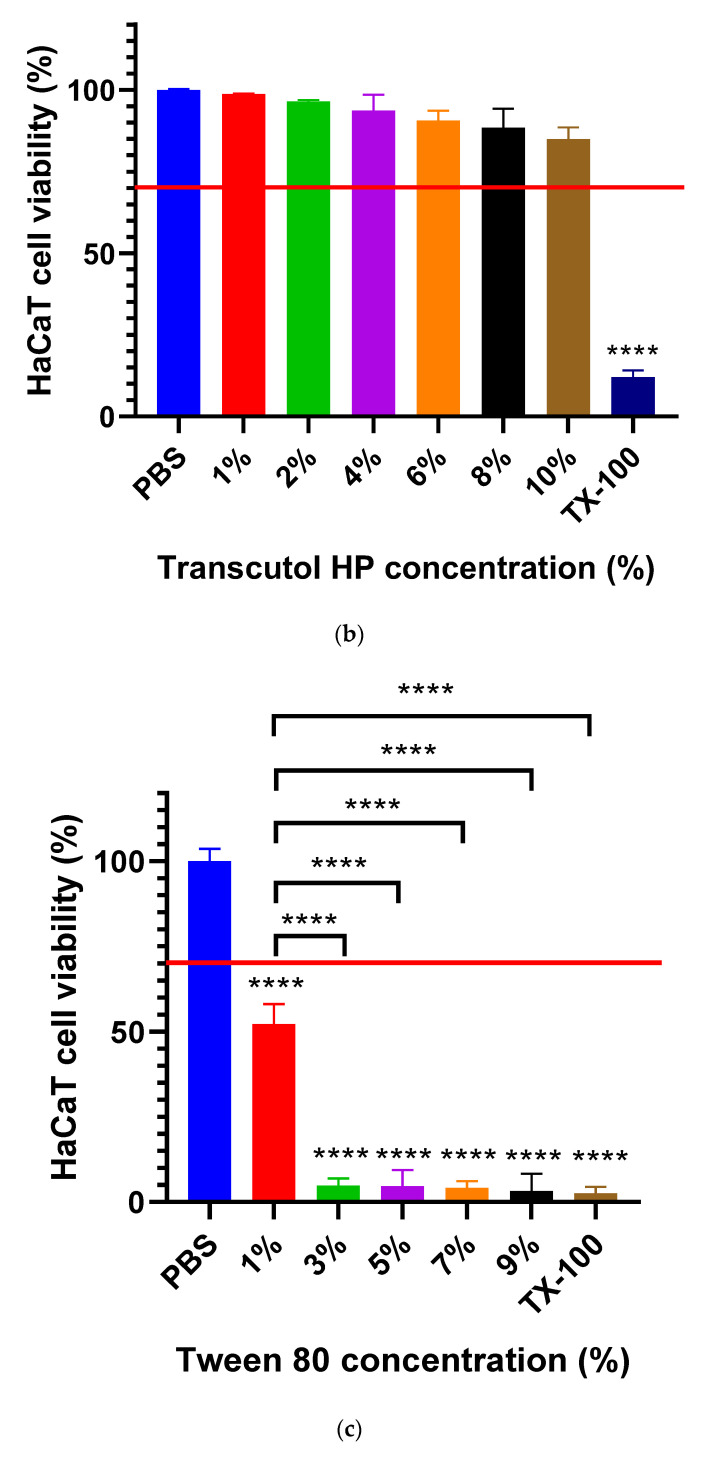
(**a**–**c**) MTT assay results of the excipients on HaCaT cell line. Cell viability values were compared to the negative control (PBS) and expressed as percentages. One-way ANOVA test was carried out for statistical analysis. Significant differences are marked with asterisks. **** indicates a statistically significant difference at *p* < 0.0001.

**Figure 3:**
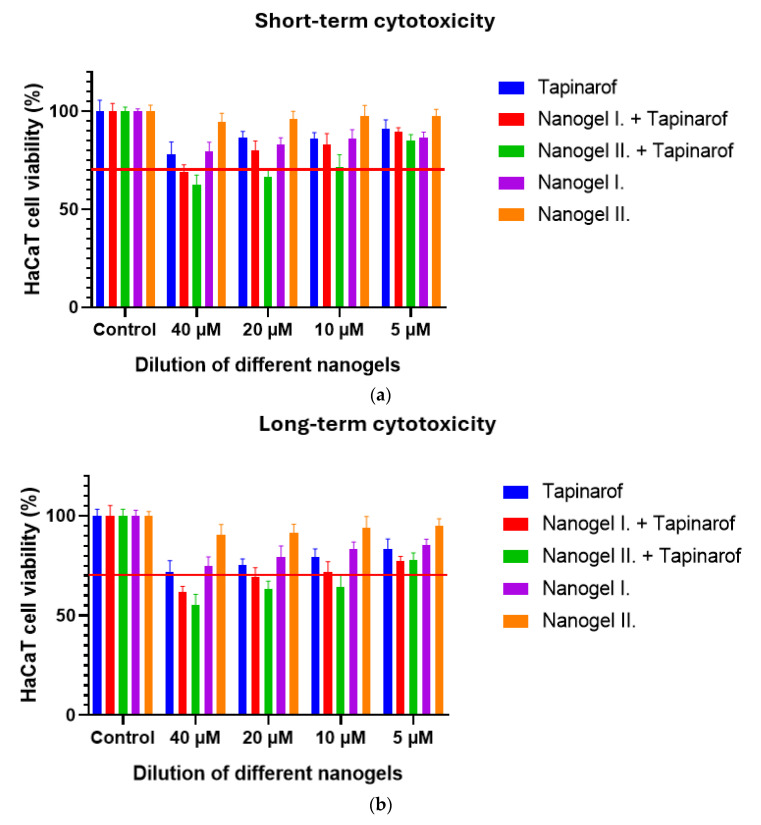
(**a**,**b**) MTT assay results of nanogels and active substances on HaCaT cell line. Cell viability values were compared to the negative control (PBS) and expressed as percentages. One-way ANOVA test was carried out for statistical analysis.

The results of the MTT test are demonstrated in Figure 2 and Figure 3. In all cases, cytotoxicity studies were conducted on HaCat cells. The experiment was performed with different excipients of the formulation to make sure that they were non-toxic for the cells. In the experiment, labrafil, transcutol HP, and tween 80 were tested and dissolved in PBS at different concentrations. In addition, the active substance and the nanogel formulations with and without tapinarof were dissolved in a 9% NaCl solution at different concentrations. During the test, triton-X 100 was chosen as a positive control and PBS as a negative control. Cell viability values were compared to the negative control and expressed as percentages. The results of the tests have shown that only tween 80 is not safe in the used concentrations. As they help the release and better penetration of the active ingredient, their use needs to be considered. All further test samples of the excipients were safe and non-toxic, as cell viability values were above 70% in all cases. The effects of tapinarof and different nanogels on HaCaT cell viability were assessed through both short-term (30 min incubation time) and long-term (24 h incubation time) studies. Results of the short-term study demonstrated that all formulations, including tapinarof alone, were not toxic on HaCaT cells. In the long-term assessments, similar values were observed. However, a slight decrease in viability was observed in some combinations after 24 h of incubation. Overall, both studies show that tapinarof and nanogel formulations are safe for use on HaCaT cells.

### 2.3. Texture Analysis

**Figure 4:**
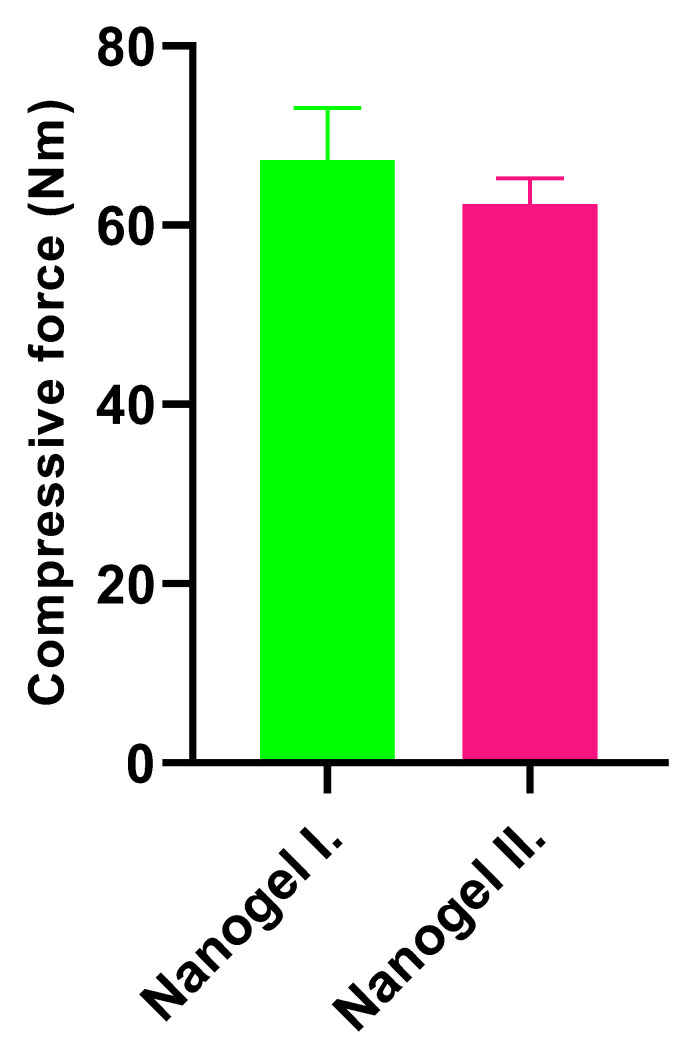
Results of the texture analysis of different formulations of nanogels with drugs incorporated. Data are expressed as means ± SD; n = 5. To compare the preparations, ordinary one-way ANOVA test was carried out.

The tested formulations were also subject to texture analysis. Figure 4 presents the required compression force to penetrate the cylinder into the preparations. As the results showed, samples did not show any resistance. Each consistency was found to be adequate for gel drug formulation due to low consistency of the gels, which helps to release the active ingredient. Their resistance was found to be low, which is a preferable factor for skin application.

### 2.4. Rheological Analysis

**Figure 5:**
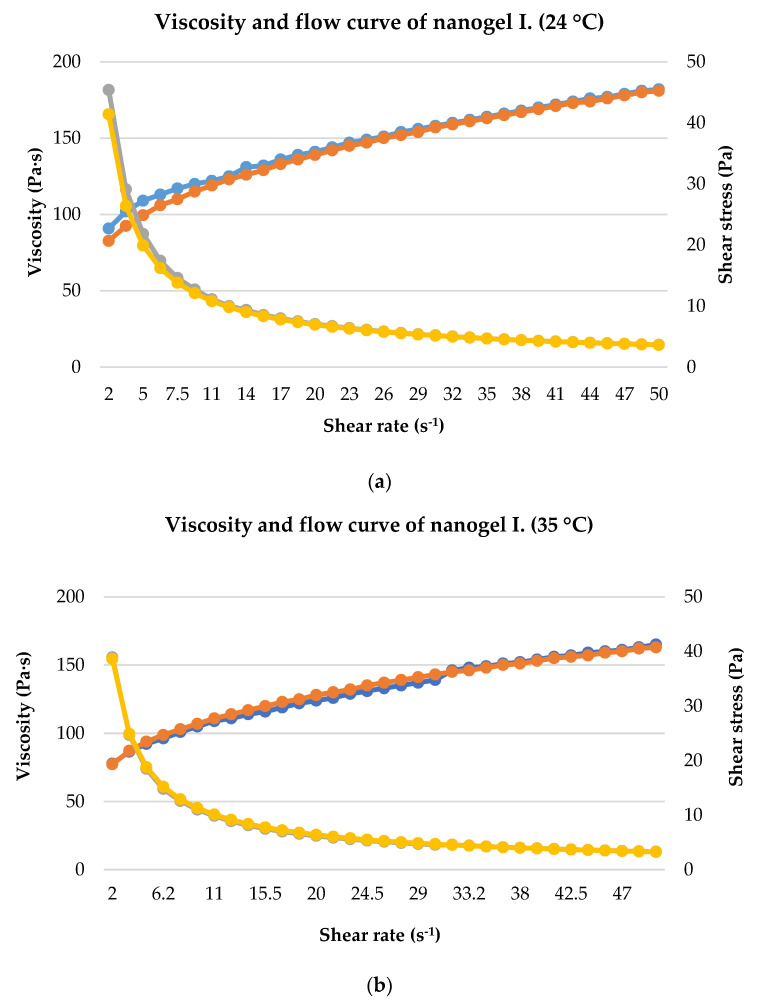

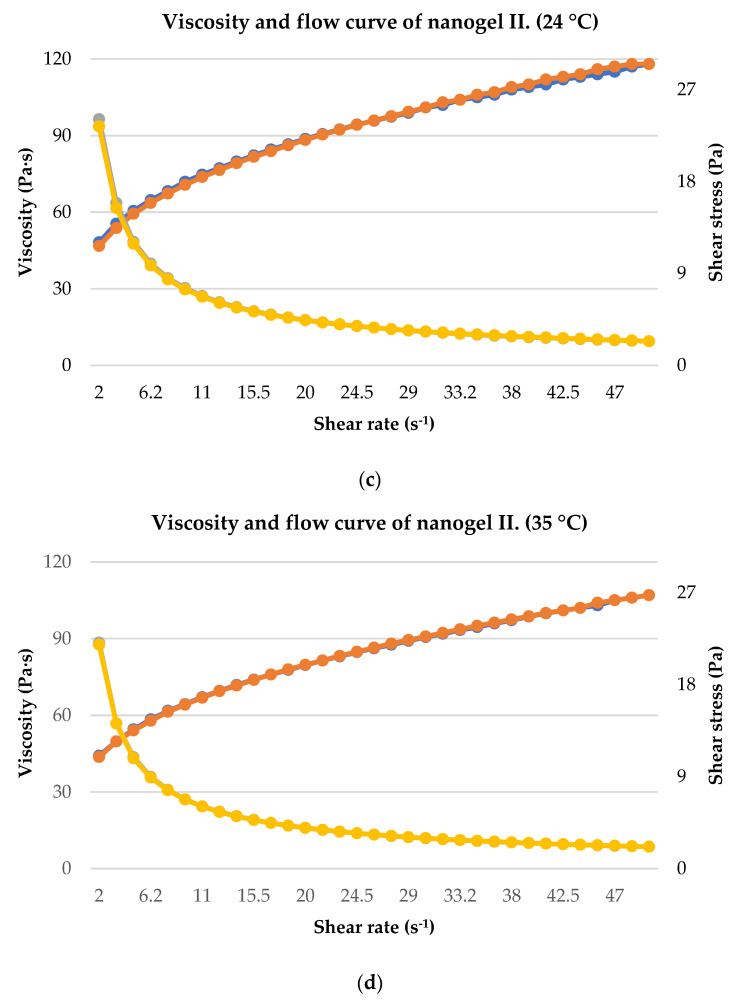
(**a**–**d**) Viscosity (upward curve) and flow curves (downward curve) of the gel samples. The results of the rheological characterization of the two formulations were presented at room temperature (24 °C) and body temperature (35 °C). Initially, shear rate was increased from 0.01 s^−1^ to 50 s^−1^, then decreased from 50 s^−1^ to 0.01 s^−1^.

The rheological characteristics were measured to assess which formulation is the most suitable for topical application, as viscosity affects the spreadability of semisolid formulations as well as the bioavailability of incorporated drug substances. To describe the rheological properties of the formulations, we used the variation of viscosity and flow, which were determined depending on the shear rate. According to the results (Figure 5), the viscosity of gels depends on the magnitude and time of the mechanical action (shear rate). Initially, the viscosity is high, then gradually decreases with increasing shear rate, showing pseudoplastic behavior. In all cases, increasing shear stress resulted in increasing shear rate, indicating that more force is required to deform the nanogels. Furthermore, the plots clearly show that nanogel I. has a higher viscosity and steeper flow curve than nanogel II. The measurements at 24 °C showed slightly higher viscosity values than the results measured at 35 °C. Thus, the viscosity of the formulations decreased with increasing temperature. During the experiment, the shear rate was first increased and then decreased to check for hysteresis effects. The upward and downward curves are practically coincident, so hysteresis loops were not typical under the experimental conditions. The internal structure of the material does not show any permanent changes under shear stress. In the absence of hysteresis, the less thixotropic behavior reflected that the formulations were able to recover their initial structure after removal of the shear force.

### 2.5. In Vitro Wound Healing Assay

**Figure 6:**
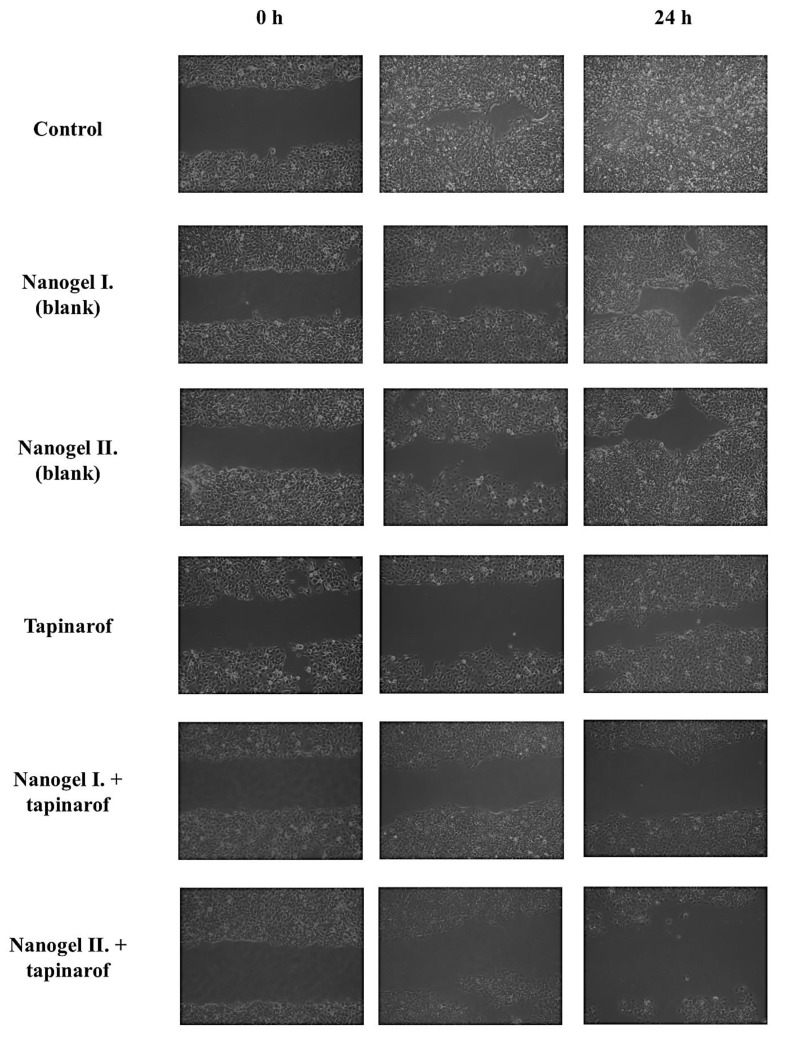
Microscopic images of the degree of closure obtained compared to those with free drugs, empty nanogels, and drug-loaded nanogels.

The effect of tapinarof-loaded nanogels on the migration of fibroblasts in cell culture was investigated using a scratch wound assay. Figure 6 presents microscopic images of the degree of closure obtained under control conditions after 24 h of treatment. The effect of the tested formulations with and without the active ingredient was examined in comparison with the closure rate obtained with free tapinarof. Cells treated with the 40 µg/mL drug-containing nanogel II. had fewer fibroblasts within the scratch area than cells treated with the empty nanogel. In the case of the blank preparations, complete closure of the scratch area was observed. The effect of the drug-loaded nanogel I. also observed cell closure in the cell culture of fibroblasts within 24 h. In conclusion, the results suggest the incorporation of nanogel II. into the nanogel increases the antiproliferative and antimigration activity of tapinarof.

### 2.6. In Vitro Release Study

**Figure 7:**
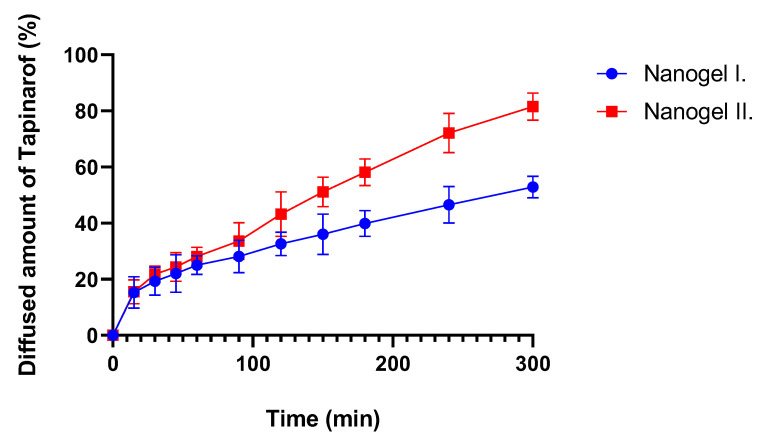
Release profile of tapinarof of different formulations.

Figure 7 shows the diffusion profile of the formulations through an isopropyl myristate-impregnated cellulose acetate membrane. The average cumulative percentage of tapinarof that permeated through the membrane was determined against time in the graphs. According to permeation studies, the drug has started to release immediately, and the dissolution curve increases constantly. It can be concluded that nanogel II. showed better performance compared to nanogel I: after 5 h, 81% of the tapinarof had leaked through the membrane, which is significantly higher than the 52% observed for nanogel I. In addition, the flux of nanogel II was 0.1935 mg/cm^2^ × h^−1^, while the flux of nanogel I was lower at 0.1255 mg/cm^2^ × h^−1^. Table 3 presents the correlation coefficient values of the compositions under two different kinetic models: zero-order and first-order. Correlation coefficients (R^2^ values) closer to 1 indicate a better fit for the kinetic model. Based on the results, both gels can be well characterized by the zero-order and first-order kinetic models.

In summary, the gels were studied using different aspects such as MTT assay and particle size determination. The nano-size of the formulations was verified by DLS. Based on the measurements, both compositions fall within the nano-size range even after gelation.

Biocompatibility tests of the active ingredient containing gels and selected excipients were performed in HaCat cell lines. The effects of tapinarof and different nanogels on cell viability were assessed through both short-term and long-term studies to present a more complete evaluation of the safety profile of the product. The results of the MTT assay showed that all the samples of gels, transcutol, and labrafil in different concentrations were considered safe. However, these results are not in accordance with the findings of Farzaneh Forouz et al. The toxicity of labrafil and transcutol were studied by two different assays (MTT and CV). Their studies found that they were less toxic than triton X-100, but they were not considered non-toxic excipients in pharmacological or toxicological evaluations [26].

In our experiment, the only exception that proved toxic was tween 80. On the other hand, several studies in the literature refute this result. Previous studies have also investigated the toxicity of the particular surfactant in comparison with others [27,28]. Caroline Maupas et al. investigated the toxicity of tween 80 and other tensides incorporated into lipid nanocapsules, which showed strong surfactant-type dependent toxicity in HaCaT cells. However, as a result, polysorbate 80 did not prove to be the most toxic. The toxicity of surfactants could be explained mainly by their amphiphilic structure [27]. However, these results are not in agreement with the studies of Maiulle T. Pacheo et al. on nanoemulsions containing sucupira oil, as the toxicity was attributed to sucupira oil and not to the applied surfactants [29]. Moreover, Katja Steine et al. evaluated it as one of the most skin-friendly surfactants as part of their study [28]. In addition, this excipient is used in numerous topical formulations [29], as well as in the marketed tapinarof-loaded ointment [20].

In the process of our work, we have tried to choose excipients that help the solubility and release of the active substance. As in the literature, non-ionic surfactants are considered to have the least irritating potential, so we decided to use them. Labrafil, tween 80 and transcutol HP are also excipients that are used as solvents or co-solvents, individually or in combination, to enhance the penetration of topical drug formulations [26]. Yue Zhang et al. looked at the solubility of tapinarof and found it to be highly soluble in tween 80 and ethanol [30]. Whereas, Jasmine Musakhanian et al. described transcutol as being able to dissolve a significant amount of hydrophilic and lipophilic material, which makes it suitable for use in hydrogels [31]. Thus, in the formulation of the gels, tapinarof was dissolved in these components.

In parallel with the experiments, texture and rheological behavior were also investigated as these parameters are related to the composition of the drugs and thus their consistency, bioavailability, and stability. In particular, the correlation of viscosity and shear stress with temperature and shear rate has a direct influence on spreadability, drug release, and consequently, drug penetration through the skin [32].

Rheological measurements showed that the viscosity of both gels decreased with increasing temperature since the viscosity of the samples was slightly lower at 35 °C than at room temperature. Similar results have been reported by Zeynep Ay Şenyiğit and co-workers who observed similar pseudoplastic behavior in their carbopol 934 based gels for dermal delivery of mupirocin [33]. In another study by Rajinikanth and co-workers, carbopol 934 based gels also revealed shear thinning behavior with a decrease in viscosity [34]. Furthermore, our results are supported by the work of Giuseppe and co-workers who observed that with an increase in temperature, the viscosity of various carbopol-containing gels decreased [35]. As viscosity decreases, we observe the effect that at body temperature the gel becomes less viscous and flows more easily. This can lead to faster release of the active ingredient as the gel spreads more easily. Nanogel II. had a slightly lower viscosity, suggesting that this one may be able to release the drug faster. Furthermore, the reduced viscosity also means that at body temperature the nanogel is easier to spread. This makes it easier to apply on the skin.

The texture analysis results showed that there was no significant difference in compression strength between the two formulations. Nanogel I. showed an average compressive force of around 65 Nm, while nanogel II. showed a slightly lower value of around 60 Nm. The tested samples did not show any resistance and as a result, their consistency was found to be adequate for gel drug formulation, which may contribute to patient compliance.

During the in vitro release studies, it was found that nanogel II. shows faster and higher tapinarof diffusion over time. After 11 min, the release of tapinarof reaches approximately 14%, while the diffusion of nanogel I. is slower and more moderate, reaching a release value of approximately 8% after 11 min. Both formulations perform stability during the experiment, but the differences between the two nanogels are clear in the release speed of tapinarof. This difference is probably due to the different ingredients used in the formulation and the rheological properties that affect the release and diffusion of the drug through the skin. Among two formulations, nanogel II. showed a better release profile, which confirms the results of the rheological studies. The release of ointments is generally slower than that of gels. This may be particularly beneficial when a faster release of tapinarof is clinically desirable for targeted treatment, for example in psoriasis.

According to the scientific literature, tapinarof has antiproliferative properties [36,37]. Yu-Qing Gu et al. have successfully demonstrated the effects of tapinarof on the proliferation of HaCaT cells were analyzed by CCK-8 assay [36]. Zhenguo Cai et al. also confirmed that tapinarof can inhibit keratinocyte hyperproliferation by reducing STAT3 phosphorylation [37]. This fact was also proved in our in vitro wound healing study. In the experiment, tapinarof by itself already showed better wound healing at the 24 h time point than empty gels. Moreover, even the drug-loaded nanogel I. did not fully inhibit cell migration, while wound closure was faster and more efficient with the drug-combined nanogel II. Fibroblast migration was successfully inhibited within the scratch area after 24 h. This is crucial because keratinocyte proliferation and differentiation are closely linked to the pathogenesis of psoriasis.

## 3. Conclusions

Tapinarof is a polyphenol of natural origin other than plant-based polyphenols, produced by the symbiotic bacteria Photorhabdus luminescens of the nematode Heterorhabditis [10,13]. Mechanism of action includes immunological regulation, skin barrier repair, and antioxidant activity [11]. It is a novel promising agent and the first and only topical treatment option for plaque psoriasis without steroids. Currently available on the market only in the form of a cream [11,20], its therapeutic efficacy might be significantly enhanced by gel-based formulations [10].

In the present study, two different formulations of carbopol-based drug delivery systems with different surfactants were formulated with the aim of boosting the anti-inflammatory effect of tapinarof and minimizing the side effects, therefore facilitating better bioavailability in the body. The experimental results show that they maintain their stable particle size after gelation and are safe and well tolerated on the HaCaT cell line, thus they are not expected to be irritating on the skin, which is a crucial factor in inflammatory skin diseases. In addition, the formulations have adequate consistency and viscosity, facilitating the controlled release of the active ingredient from the formulations for more effective drug delivery. Based on wound healing studies, these nanoformulations enhance the antiproliferative and antimigratory activity of the drug and our results suggest that they could be a promising addition to future psoriasis therapies.

## 4. Materials and Methods

### 4.1. Materials

HaCat cell line was supplied by Cell Lines Service (CLS, Heidelberg, Germany). Culturing flasks and 96-well plates were obtained from Corning (Corning, New York, NY, USA). Dulbecco’s Modified Eagle’s Medium (DMEM), phosphate-buffered saline (PBS), trypsin-EDTA, 3-(dimethylthiazol-2-yl)-2,5-diphenyltetrazolium bromide (MTT paint), tapinarof, triacetin, Tween 80 were purchased from Sima Aldrich (St. Gallen, Switzerland). Ethanol 96%, glycerin 85%, and oleic acid were obtained from Molar Chemicals Kft. (Budapest, Hungary). We purchased kollisolv PEG 400 and kolliphor EL from BASF Company (Ludwigshafen, Germany). Carbopol 940 and carbopol 934 were obtained from B.F. Goodrich Chemical Company (Charlotte, North Carolina). Triethanolamine was purchased from VWR Chemicals. Labrafil M and transcutol HP were kindly gifted from Gattefossé (Lyon, France).

### 4.2. Preparation of Nanogels

Initially, the amount of tapinarof was successfully incorporated into the formulation by dissolving it in transcutol and ethanol. The surfactants (labrafil, triacetin, tween 80, kolliphor, and oleic acid) were used in combination with surfactants (transcutol, ethanol, and PEG 400) to improve stability and texture and help the active ingredient to penetrate through the skin. The excipients were combined using a magnetic stirrer (Radelkis, Budapest, Hungary) to produce the oil phase. The rotation of the magnetic stirrer was 400 rpm for 15 min. This was followed by the addition of the aqueous phase to produce SNEDDS and vortexed for 5 min. Carbopol was added as a gelling agent. The preparation of carbopol gels was performed by dispersing the required amount of carbopol powder in a small amount of purified water and leaving for 24 h to achieve uniform swelling. Glycerin was added as a humectant to nanogel I. The SNEDDS were then added dropwise to the gel matrix while magnetic stirrer was used at 500 rpm. To achieve the nano-size range, UP200Ht (200W, 26kHz) manual homogenizer was applied before gelation. The pH of the gels was approached to the pH of the skin by adding a few drops of triethanolamine, thus creating a homogeneous structured nanogel [16,21]. The amounts of all materials used are listed in Table 4 and Table 5.

### 4.3. Droplet Size Distribution

To analyze particle size Malvern Zetasizer Nano ZSP (Malvern Panalytical; Malvern, UK) was used to show tapinarof-loaded nanogels do not lose their stable particle size even after gelation. Before analysis, preparations were 1000 times diluted with purified water. The samples were allowed to equilibrate for 5 min at 25 °C before performing 3 measurements consisting of 13 runs each [38].

### 4.4. Cell Viability Study

The safety of the excipients and the active substance used for SNEDDS was tested by MTT assay. Cell viability was carried out on HaCat cell lines to verify the cytotoxicity. HaCat cells can perfectly represent human skin as they are human immortalized keratinocytes. The cell line was maintained by weekly passages in Dulbecco’s Modified Eagle’s Medium. Cells were seeded in 96-well plates at a density of 10,000 cells/well to carry out the experiment. When the cells had completely colonized the well membrane, the culture medium was first removed. Then the dilutions of the excipients were applied to the cells. This was followed by a 30 min incubation for short-term studies and 24 h for long-term studies. After that, the dilutions were removed, and MTT staining solution (tetrazolium bromide) was added to the cells at a concentration of 5 mg/mL. The cells were kept incubated for 3 h. Viable cells convert the yellow water-soluble tetrazolium bromide into purple formazan precipitates. At the end of incubation, the formazan precipitate was dissolved in isopropanol: hydrochloric acid (25:1). The absorbance of the samples was then determined using a spectrophotometer (Fluostar Optima). The result is directly proportional to the number of viable cells [39,40,41,42].

### 4.5. Texture Analysis

The compression test was conducted, and the resistance of nanogels was measured using a CT3 Texture Analyzer (Brookfield, Middleboro, MA, USA). For the analyses, the vessel filled with the given composition was placed approximately 5,50 cm below the probe of the texture analyzer. Before the measurement, the probe was lowered onto the sample surface at a speed of 1 mm/s. After reaching the surface, the probe was penetrated to a depth of 10.0 mm at 0.50 mm/s, and the force exerted on the probe was measured. The force applied to the sensor was recorded using Texture Pro CT software version 1.3 (Brookfield Engineering Laboratories, MA, USA). The texture analyzer was equipped with a TA5 cylinder-type probe (12.7 mm diameter and 35 mm long) during the test. The compression test was performed in normal mode with the following parameters: speed (0.50 mm/s), trigger load (4.0 g), and target (10.0 mm). Compression experiments were performed at room temperature (25 °C). All measurements were repeated three times. The means and standard deviations were determined [40,41,43,44].

### 4.6. Rheological Analysis

Rheological measurements were also carried out to determine rheological properties using RheolabQC rotational rheometer. The viscosity and flow curves of the preparations were evaluated using RheoPlus Rheometer Software (32 V3.10 21003407-33024). The viscosity curves of the preparations were evaluated by rotational tests with controlled shear rate. 30 g of the preparation was loaded into the cup of the concentric cylindrical measuring system of the rheometer. Both viscosity and flow were measured for each gel at room temperature (24 °C) and body temperature (35 °C). The shear rate was first increased from 0.01 s^−1^ to 50 s^−1^ and then decreased again to 0.01 s^−1^ to check for hysteresis effects [44,45].

### 4.7. In Vitro Wound Healing Assay

Fibroblast cells were seeded at a density of 1 × 10^4^ cells in a 12-well chamber (Thermo SC NUNC, Roskilde, Denmark). Confluent monolayers were synchronized in DMEM containing 0.5% FBS for 2 h. Using a standard 200 μL pipette tip, a 500–800 μm wide strip was removed from the cells through the well. The wound culture wells were washed with PBS to remove non-adherent cells. In addition, monolayers were treated with free tapinarof dissolved in transcutol HP or blank gels or gels containing tapinarof (5, 10, 20, 40 μg/mL). The wound area was observed and photographed at 0 and 24 h using an optical microscope (Axiovert, ZEISS, Jena, Germany) [46].

### 4.8. In Vitro Release Study

In vitro release of tapinarof from the different concentrations of the gels was determined by using a Franz diffusion chamber apparatus. A membrane was placed between the acceptor and donor phases. The concentration profile of the test substance is determined by sampling at predefined time points. Samples of 500 mg weight were applied to an artificial cellulose acetate membrane (0.5 µm pore size) as donor phase, and a pH = 5 buffer was chosen as acceptor phase to imitate the pH of the skin. The receptor phase was maintained at 32 °C. The membrane was pretreated with isopropyl myristate to characterize the lipophilic property of the skin. The rotation of the magnetic stirrer was 450 rpm. Samples were taken from the receptor phase after 15, 30, 60, 90, 120, 150, 180, 240, and 300 min. The tapinarof content was measured by spectrophotometry. Data were fitted to zero- and first-order kinetics (Table 3). The mathematical model for in vitro release is reported in Table 6 [39,40,41,42].

*Flux* was calculated using the following Equation (1) [42]:

(1) $$\mathrm{Flux}=\frac{Q}{t}$$

where *Q* is the amount of drug released per unit area (mg/cm^2^) and diffusion time (*t*).

### 4.9. Statistical Analysis

Data were analyzed with GraphPad Prism 6 and presented as means ± SD. Comparison of the groups in MTT assays and texture analysis were performed with one-way ANOVA test. Significant differences in the figures are signed with asterisks. Differences were regarded as significant when *p* < 0.05. All experiments were performed at least in triplicate [39].

## Figures and Tables

**Table 1:** Size distribution of gel I. after gelation.

Time (min)	Amp (%)	Z-Average (d/nm)	PdI
2	50	275.6	0.282
3	50	172.8	0.210

**Table 2:** Size distribution of gel II. after gelation.

Time (min)	Amp (%)	Z-Average (d/nm)	PdI
2	50	151.1	0.169

**Table 3:** Correlation coefficient values of the preparations.

Kinetic Model		
Composition	Zero	First
Nanogel I.	0.9892	0.978
Nanogel II.	0.994	0.998

**Table 4:** Composition of different SNEDDS.

Composition	SNEDDS I.	SNEDDS II.
Labrafil	6%	-
Triacetin	3%	-
Tween 80	5%	8.75%
Transcutol HP	15%	-
Ethanol	5%	26.25%
Kolliphor	5%	-
PEG 400	5%	-
Oleic acid	-	10%
Purified water	56%	55%

**Table 5:** Composition of the formulated nanogels.

Composition	Nanogel I.	Nanogel-Tap I.	Nanogel II.	Nanogel-Tap II.
Tapinarof	-	1%	-	1%
SNEDDS	33.3%	33.3%	50%	50%
Glycerin	5%	5%	15%	-
Carbopol 940	0.5%	0.5%	5%	-
Carbopol 934	-	-	0.5%	0.5%

**Table 6:** Mathematical model of drug release profiles.

Model	Equations	Graphic
Zero-order	*Q_t_ = Q_0_ + k_0_t*	The graphic of the drug-dissolved fraction versus time is linear.
First-order	$Q_{t}=Q_{0}\cdot x\cdot e^{-k_{1}t}$	The graphic of the decimal logarithm of the released amount of drug versus time is linear.

where *Q_0_* is the initial amount of drug; *Q_t_* is the amount of drug remaining at time *t*; *Q_t_/Q_0_* is the fraction of drug released at time *t; k_0_* and *k_1_* are the kinetic constants.

## Data Availability

The original contributions presented in this study are included in the article; further inquiries can be directed to the corresponding author.
